# High rate of successful treatment outcomes among childhood rifampicin/multidrug-resistant tuberculosis in Pakistan: a multicentre retrospective observational analysis

**DOI:** 10.1186/s12879-021-06935-6

**Published:** 2021-12-04

**Authors:** Farah Naz, Nafees Ahmad, Abdul Wahid, Izaz Ahmad, Asad Khan, Muhammad Abubakar, Shabir Ahmed Khan, Amjad Khan, Abdullah Latif, Abdul Ghafoor

**Affiliations:** 1 Department of Pharmacy Practice, Faculty of Pharmacy and Health Sciences, University of Balochistan, Quetta, Pakistan; 2 Department of Biology, Syed Babar Ali School of Science and Engineering, Lahore University of Management Sciences, Lahore, Pakistan; 3 Pakistan Centre of Excellence in Vaccinology & Biotechnology, University of Balochistan, Quetta, Pakistan; 4 Department of Pharmacy, Quaid-i-Azam University, Islamabad, Pakistan; 5 National TB Control Program, Islamabad, Pakistan

**Keywords:** Childhood, Ethambutol, Females, Rifampicin resistant-TB, MDR-TB

## Abstract

**Background:**

There was a complete lack of information about the treatment outcomes of rifampicin/multidrug resistant (RR/MDR) childhood TB patients (age ≤ 14 years) from Pakistan, an MDR-TB 5th high burden country. Therefore, this study evaluated the socio-demographic characteristics, drug resistance pattern, treatment outcomes and factors associated with unsuccessful outcomes among childhood RR/MDR-TB patients in Pakistan.

**Methods:**

This was a multicentre retrospective record review of all microbiologically confirmed childhood RR/MDR-TB patients (age ≤ 14 years) enrolled for treatment at seven units of programmatic management of drug-resistant TB (PMDT) in Pakistan. The baseline and follow-up information of enrolled participants from treatment initiation until the end of treatment were retrieved from electronic nominal recording and reporting system. World Health Organization (WHO) defined criterion was used for deciding treatment outcomes. The outcomes of “cured” and “treatment completed” were collectively grouped as successful, whereas “death”, “treatment failure” and “lost to follow-up” were grouped together as unsuccessful outcomes. Multivariable binary logistic regression analysis was used to find factors associated with unsuccessful outcomes. A p-value < 0.05 reflected statistically significant findings.

**Results:**

A total of 213 children RR/MDR-TB (84 RR and 129 MDR-TB) were included in the study. Majority of them were females (74%), belonged to the age group 10–14 years (82.2%) and suffered from pulmonary TB (85.9%). A notable proportion (37.1%) of patients had no history of previous TB treatment. Patients were resistant to a median of two drugs (interquartile range: 1–4) and 23% were resistant to any second line anti-TB drug. A total of 174 (81.7%) patients achieved successful treatment outcomes with 144 (67.6%) patients being cured and 30 (14.1%) declared treatment completed. Among the 39 (18.3%) patients with unsuccessful outcomes, 35 (16.4%) died and 4 (1.9%) experienced treatment failure. In multivariable analysis, the use of ethambutol had statistically significant negative association with unsuccessful outcomes (odds ratio = 0.36, p-value = 0.02).

**Conclusions:**

In this study, the WHO target of successful treatment outcomes (≥ 75%) among childhood RR/MDR-TB patients was achieved. The notable proportion of patients with no history of previous TB treatment (37.1%) and the disproportionately high number of female patients (74%) respectively stress for infection control measures and provision of early and high quality care for female drug susceptible TB patients.

**Supplementary Information:**

The online version contains supplementary material available at 10.1186/s12879-021-06935-6.

## Background

The incidence and spread of multidrug resistant tuberculosis (MDR-TB) defined as “TB caused by strains of *Mycobacterium tuberculosis* (MTB) concurrently resistant to both rifampicin (R) and isoniazid (H)” are threatening to the successful control and eradication of TB [1]. It is estimated that out of 10 million TB cases in 2019, a total 3.3% of the new and 18% of the previously treated TB cases had MDR/R resistant (RR) TB [2]. In 2019, there were approximately 465,000 (range 400,000–535,000) incident cases of RR-TB; out of which 78% had MDR-TB [2]. Being resistant to the powerful and safe first line anti-TB drugs (FLD) i.e. R and H, these patients are treated with a long, less effective and toxic regimen mainly comprised of multiple second-line anti-TB drugs (SLD) [2]. This results in comparatively poor treatment outcomes in these patients [1]. The global treatment success rates of 2017 cohorts of MDR/RR-TB and drug susceptible TB were respectively 56% and 85% [2].

Similar with other forms of TB, drug resistant-TB (DR-TB) affects people of all age groups including children (age ≤ 14 years) [3]. It has been estimated that each year approximately 25,000–32,000 children develop MDR-TB, which makes 3% of all childhood TB cases [4]. Because of children incapability to expectorate sputum, paucibacillary nature of the disease, problems in obtaining specimens for culture and drug susceptibility testing (DST), and nonspecific symptoms, the diagnosis of childhood TB and DST are challenging tasks. Consequently, childhood TB including DR-TB has suffered a historical neglect and has not been a priority of national TB programs (NTP) throughout the world [3, 5]. Although children suffering from MDR-TB have a diverse spectrum of disease, differences in metabolism of anti-TB drugs, different range of adverse events and healthcare needs than adults [6–8], still they are treated with the same treatment regimens as adult MDR-TB patients. The previously published very few individual cohorts of childhood MDR-TB patients have reported variable rates of successful treatment outcomes (range 62–92%) [6, 9, 10]. A systematic review and meta-analysis of 1413 childhood MDR-TB patients has reported a pooled treatment success rate of 73% in developing and 87% in developed countries [11]. An individual patients’ data meta-analysis of 975 childhood MDR-TB patients has reported a treatment success rate of 78% [12]. Variable treatment success rate among childhood DR-TB patients could be due to different proportion of comorbidities, disease severity, drug resistance patterns [6, 9–12] and different DR-TB treatment regimens used over the last decade. The conventional longer treatment regimen (LTR) was introduced by WHO in 2011. For RR/MDR-TB patients without resistance to any SLD, the LTR comprised of at least 8 months treatment with *amikacin (Am)/kanamycin (Km)/capreomycin (Cm)* + *levofloxacin (Lfx)* + *ethionamide (Eto)* + *cycloserine (Cs)* + *pyrazinamide (Z)* and 12 months treatment with *Lfx* + *Eto* + *Cs* + *Z.* For patients with resistance to any SLD, it was recommended to add *para-amino salicylic acid (PAS)* to the abovementioned regimen [13]. In order to overcome the disadvantages of low treatment success rate, high incidence of clinically significant adverse events, prolonged treatment duration and high cost associated with LTR [1], in 2016 WHO recommended a shorter treatment regimen (STR). It comprised of treating MDR/RR-TB patients for 4–6 months with *Km* + *moxifloxacin (Mfx)* + *prothionamide (Pto)* + *clofazimine (Cfz)* + *Z* + *ethambutol (E)* + *high dose H* followed by 5 months treatment with Mfx + *Cfz* + *Z* + *E* [14]*.* However, the limited applicability of STR due to strict eligibility criteria for patients being treated with STR [15] resulted in the introduction of updated regimens for the treatment of MDR/RR-TB patients in 2020 [16]. These regimens comprised of (i) shorter all oral *bedaquiline* containing regimen i.e. treatment for 4–6 months with *bedaquiline* and 6 months with *Lfx/Mfx* + *Cfz* + *Z* + *E* + *High dose H* followed by 5 months of *Lfx/Mfx* + *Cfz* + *Z* + *E* (ii) various *bedaquiline* containing LTRs and (iii) *bedaquiline*, *pretomanid and linezolid* (BPaL) containing regimen.

Unluckily, Pakistan is currently MDR-TB 5th high burden country, where the programmatic management of DR-TB (PMDT) was started way back in 2010 and at present there are 33 functional PMDT units in the country. Investigating the routine management and treatment outcomes of a group of patients is a conventional and effective way of assessing the program’s efficacy. In Pakistan, the previously published multiple cohorts of MDR-TB patients have reported a variable treatment success rate (range: 40.5–83.7%) [17–22]. However, there was a lack of information regarding socio-demographic characteristics, drug resistance pattern, treatment outcomes and factors associated with unsuccessful outcomes among childhood RR/MDR-TB patients from Pakistan. Therefore, the present study was carried out with the objective to fill the abovementioned gap.

## Methods

### Study design, setting and duration

The present study was a retrospective observational investigation carried out at the following seven PMDT units (i) Lady Reading Hospital (LRH), Peshawar (ii) Nishter Hospital Multan (NHM) (iii) Rawalpindi Leprosy Hospital, Rawalpindi (iv) Jinnah Hospital, Lahore (v) Saidu Teaching Hospital, Swat (vii) District Head Quarter Hospital, Faisalabad and (viii) Sheikh Zaid Hospital, Rahim Yar Khan. All microbiologically confirmed childhood RR/MDR-TB patients (age ≤ 14 years) enrolled at the abovementioned sites between 2010 to 31st May 2019 in LRH, 2012 to 31st May 2019 in NHM, and 2017 to 31st May 2019 in all other centers were included in the current study irrespective of site of disease and the type of treatment regimen they received. The baseline and follow-up information of enrolled participants from treatment initiation until the end of treatment were retrieved from electronic nominal recording and reporting system (ENRS).

### Diagnosis and treatment of MDR/RR-TB

The diagnosis and management of MDR/RR-TB at PMDT sites in Pakistan with both longer and shorter treatment regimens have been previously described elsewhere [17–22]. In summary, at these sites, DR-TB was diagnosed and managed in compliance with the recommendations of guidelines for the management of DR-TB published and disseminated by NTP [23]. At PMDT sites, two sputum samples of every presumed DR-TB patient were collected. If the patients were unable to produce sputum, they were either subjected to sputum induction or their bronchoalveolar lavage or gastric aspirates were taken by using standard methods [24]. The samples taken were initially assessed for MTB, R and H resistance by direct sputum smear microscopy using Ziehl–Neelsen staining, Xpert MTB/Rif (Cepheid, Sunnyvale, CA, United States) and line probe assay (LPA) (2018 onward) [17–22]. After positive finding for MTB and RR, treatment was initiated with an empirical MDR-TB treatment regimen recommended by NTP guidelines. Meanwhile, the patient’s sputum samples were referred to national or provincial reference laboratories for phenotypic culture and DST. At the reference laboratories, DST against FLD and SLD were carried out by Agar proportion method on enriched Middlebrook 7H10 medium (BBL; Beckton Dickinson, Sparks, MD, United States) at the concentrations given elsewhere [15, 17, 21, 25, 26]. DST for Z at a concentration of 100 μg/mL was carried out by using BACTEC Mycobacterial Growth Indicator Tube (MGIT, BD, Sparks, MD, United States) [15, 17, 21, 25, 26]. After the availability of DST results, prior to 2018 each childhood MDR/RR-TB patient was put on individualized longer treatment regimen (LTR) based on his/her DST results. The LTR for childhood MDR/RR-TB patient with no resistance to any SLD was comprised of *Am/Km/Cm* + *Lfx* + *Eto* + *Cs* + *Z*. In case of resistance to any SLD, *PAS* was added to the abovementioned regimen. Childhood MDR/RR-TB patients with LTR were treated for at least 20 months with a minimum of 18 months after sputum culture conversion (SCC) defined as “two successive negative sputum cultures taken at least 1 month apart after a positive culture” [21]. The intensive phase of LTR which included an injectable SLD lasted for at least 8 months with a minimum of 6 months post SCC. After 2017, eligible childhood MDR/RR-TB patients were treated with STR and those who were not eligible for STR were treated with LTR. The eligibility criteria for treatment with STR at these sites has been given elsewhere [22]. The STR was comprised of 4–6 months of *Am* + *Cfz* + *Mfx* + *Z* + *E* + *high dose H* followed by 5 months of *Cfz* + *Mfx* + *Z* + *E* [22]. In the current cohort, treatment of eligible childhood patients with *bedaquiline, linezolid* and *delamanid* containing regimen at various PMDT centres was initiated in 2017. In order to prevent peripheral neuropathy, vitamin B6 was received by all patients. All childhood MDR/RR-TB patients were treated as outpatients and free of cost. Trained treatment supporters and home visits by home DOTS linkage facilitator ensured the patients’ adherence with their treatment regimen. At PMDT units, on each monthly visit, patients of age ≥ 10 years who were able to communicate were counselled and psychologically assessed by a clinical psychologist using diagnostic and statistical manual of mental disorders, fourth edition (DSM-IV TR) criteria for depression and Hamilton Depression Rating (HDR) scale. On HDR scale, the scores of 0–7 were considered as being normal, 8–16 suggested mild depression, 17–23 moderate depression and scores over 24 were indicative of severe depression [27, 28]. Furthermore, monthly food ration and travelling fare were given to each patient and his/her treatment supporter.

### Data collection

All PMDT sites share DR-TB patients’ with NTP through ENRS on monthly basis. ENRS is actually a combined excel sheet containing information about the patients’ sociodemographic characteristics, history of TB treatment, regimen, outcomes and previous TB treatment centre, comorbidity status, history of any SLD used, results of Xpert MTB/Rif and LPA, monthly weight, sputum smear microscopy and culture results, DST results, treatment regimen for DR-TB and end TB treatment outcomes. The abovementioned data were retrieved from ENRS through a purpose designed data collection form. Weight for age chart given by Centre for Disease Control and Prevention was used to assess children weight for age. On the basis of Centers for Disease Control and Prevention data table of weight-for-age charts, children with a body weight < 5th percentile at the baseline visit were categorized as underweight [5, 29]. Treatment outcomes of patients were based on definitions given in WHO and NTP guidelines [14, 16, 23]. The outcomes of “cured” and “treatment completed” were grouped together as “successful treatment outcomes”, whereas, “death”, “lost to follow up (LTFU)” and “treatment failure” were grouped together as unsuccessful treatment outcomes.

### Statistical analysis

Statistical Package for Social Sciences (SPSS) version 23 was used for data analysis. Categorical data were displayed as frequencies and percentages, whereas, continuous data were presented as mean ± standard deviation (SD) and median with ranges. Multivariable binary logistic regression (MVBLR) analysis was used to find final factors associated with unsuccessful treatment outcomes. After checking for correlations, variables which had an association with unsuccessful treatment outcomes at a p-value of < 0.2 were included in MVBLR analysis. If independent variables had high correlation with each other (Tolerance value < 0.1 and/or Variance inflation factor = 10), one of them was excluded from the final model. Inclusion of independent variables in the univariate analysis was based on published literature, their clinical relevancy with treatment outcomes in DR-TB and suggestions from the clinical team [6, 9–12, 17–22]. Discrimination power of the final model for predicting unsuccessful treatment outcomes was evaluated by using Receiver Operating Characteristic Curve (ROC) analysis [19]. Findings with a p-value < 0.05 were considered statistically significant.

## Results

### Patients’ baseline socio-demographic characteristics and drug resistance pattern

In the current study, a total of 213 childhood MDR/RR-TB patients were included. Among them, 129 (60.6%) suffered from MDR-TB and 84 (39.4%) from RR-TB. Out of 84 RR-TB patients, 64 patients had pulmonary and 20 had extra-pulmonary TB. Among the 64 pulmonary RR-TB patients, 22 had diagnostic positive culture results and were phenotypically confirmed to have RR-TB. Of the remaining 42 pulmonary RR-TB patients, 17 diagnostic cultures were negative and 25 were contaminated. Of the 20 extra-pulmonary RR-TB patients, diagnostic culture were not performed for 15 patients whereas 5 had negative culture results. At baseline visit, the mean age and weight of patients were respectively 11.35 ± 3.28 years [median = 12 years, interquartile range (IQR) 10–14 years] and 28.54 ± 9.73 kg (median = 30 kg, IQR 22–34 kg). Based on CDC weight for age chart, a total of 117 (54.9%) patients had a baseline body weight of < 5th percentile. Majority of patients were females (n = 160, 74%), belonged to age group 10–14 years (n = 175, 82.2%), previously been treated for TB (n = 125, 58.7%), had not received any SLD (n = 205, 96.2%), suffered from pulmonary TB (n = 183, 85.9%) and had no co-morbidity (n = 198, 93.2%) (Table 1). Upon cross-tabulation, we found that 140/160 (87.5%) female childhood patients were 10–14 years old.

**Table 1:** Patients’ baseline socio-demographic, clinical and microbiological characteristics

Variables	No. (%)
Patients enrolled in each PMDT site	
Lady Reading Hospital, Peshawar	102 (47.9)
Rawalpindi Leprosy Hospital Rawalpindi	43 (20.2)
Nishter Hospital, Multan	41 (19.2)
Jinnah Hospital, Lahore	8 (3.8)
Saidu Teaching Hospital, Swat	7 (3.3)
District Head Quarter Hospital, Faisalabad	7 (3.3)
Sheikh Zaid Hospital, Rahim Yar Khan	5 (2.3)
Age (years)	
< 5	14 (6.6)
5–9	24 (11.3)
10–14	175 (82.2)
Gender	
Female	160 (75.1)
Male	53 (24.9)
Baseline body weight	
Normal	96 (45.1)
Below normal	117 (54.9)
History of TB treatment	
No	79 (37.1)
Yes	125 (58.7)
Unknown	9 (4.2)
Previous TB treatment regimen	
New patients	79 (37.1)
Category-I	97 (45.5)
Category-II	28 (13.1)
Unknown	9 (4.2)
Previous use of second line anti-TB drugs	
No	205 (96.2)
Yes	8 (3.8)
Co-morbidity	
No	198 (93.0)
Yes	15 (7.0)
Type of co-morbidity	
Diabetes mellitus	3
Depression	11
Epilepsy	1
Type of drug-resistant TB	
Multidrug resistant TB	129 (60.0)
Rifampicin resistant TB	84 (39.4)
Site of disease	
Pulmonary TB	183 (85.9)
Extra-pulmonary TB	26 (12.2)
Both	4 (1.9)
Baseline smear grading	
Negative	46 (21.6)
Scanty (1–9 AFB/100 HPF)	15 (7.0)
+ 1 (10–99 AFB/100 HPF)	76 (35.7)
+ 2 (1–9 AFB/HPF)	42 (19.7)
+ 3 (> 9 AFB/100 HPF)	34 (16.0)

*AFB* acid fast bacilli, *HPF* high power field, *PMDT* Programmatic management of drug resistant TB, *TB* tuberculosis

The study participants were resistant to a median of two drugs (IQR 1–4 drugs). The patients’ drug resistance pattern is given in Table 2.

**Table 2:** Patients’ baseline drug resistance pattern

Variables	Frequency (%)
Number and pattern drug resistance	
One	83 (39.0)
R	83 (39.0)
Two	32 (15.0)
RH	31
R + Eto	1
Three	37 (17.4)
RH + FQ	14
RHZ	12
RHS	5
RHE	4
RH + SLI	2
Four	21 (9.9)
RHEZ	9
RHZ + FQ	5
RHZS	3
RHES	2
REZS	1
RHZ + SLI	1
Five	25 (11.7)
RHEZS	14
RHEZ + FQ	8
RHES + FQ	2
RHZS + Eto	1
Six	12 (5.6)
RHEZS + FQ	12
Seven	3 (1.4)
RHEZS + FQ + Eto	3
Resistance to R	213 (100)
Resistance to H	129 (60.6)
Resistance to E	54 (25.4)
Resistance to Z	67 (31.5)
Resistance to S	48 (21.9)
Resistance to all five FLDs	29 (13.6)
Resistance to any SLDs	50 (23.0)
Resistance to FQs	45 (21.1)
Resistance to any SLI	3 (1.4)
Resistance to Eto	5 (2.3)

*E* ethambutol, *Eto* ethionamide, *FLDs* first line anti-TB drugs, *FQs* fluoroquinolones, *H* isoniazid, *R* rifampicin, *S* streptomycin, *SLDs* second-line anti-TB drugs, *SLI* second line injectable, *Z* pyrazinamide

### Treatment regimen

In the current cohort a total of 198 (93.0%) patients were treated with LTR. The STR was received by only 15 (7.0%) patients (Table 3). A total 8 (3.8%) patients were on SLI free regimen (7 were treated with FQ + Eto + Cs + Z + Lzd/FQ + Eto + CS + Z + Lzd and 1 was on FQ + Eto + Cs + Z + Lzd + H + E/FQ + Eto + CS + Z + Lzd + H + E). Furthermore, 6 and 4 patients respectively received *bedaquiline* and *delamanid* containing regimen. All 6 patients who received bedaquiline containing regimen were ≥ 13 years old, suffered from MDR-TB and had no comorbidity, 5 among them were females, 4 patients had no history of TB treatment and 5 were resistant to FQs.

**Table 3:** Treatment regimens

Treatment regimen	No. (%)
Longer treatment regimen	198 (93.0)
SLI + FQ + Eto + Cs + Z/FQ + Eto + Cs + Z	68 (31.9)
SLI + FQ + Eto + Cs + PAS + Z/FQ + Eto + Cs + PAS + Z	55 (25.8)
SLI + FQ + Eto + Cs + Z + E/FQ + Eto + Cs + Z + E	30 (14.1)
SLI + FQ + Eto + Cs + PAS + Z + E/FQ + Eto + Cs + PAS + Z + E	10 (4.7)
Others^a^	35 (16.4)
Shorter treatment regimen	15 (7.0)
Am + Z + FQ + Eto + Cfz + high dose H + E/Z + FQ + Cfz + E	15 (7.0)

*Cs* cycloserine, *E* ethambutol, *Eto* ethionamide, *FQ* fluoroquinolones, *Km* kanamycin, *PAS* para-amino salicylic acid, *SLI* second-line injectable (amikacin/kanamycin/capreomycin), *Z* pyrazinamide

^a^Given in Additional file 1: Table S1

### Sputum culture conversion

Out of 213 patients included in the current study, 187 (87.9%) suffered from pulmonary TB (183 PTB and 4 both PTB and extra-PTB). Of 187 PTB patients, diagnostic sputum culture results were positive for 151 patients. Among these 151 patients, 129 (85.4%) achieved SCC. The median time to SCC was 2 months (IQR: 1–3 months). Of 129 patients who achieved SCC, 95 (74.8%) achieved it in initial two months of treatment.

### Treatment outcomes and factors associated with unsuccessful outcome

A total of 174 (81.7%) patients achieved successful treatment outcomes with 144 (67.6%) patients being cured and 30 (14.1%) declared treatment completed. Among the 39 (18.3%) patients with unsuccessful outcomes, 35 (16.4%) died and 4 (1.9%) experienced treatment failure. None of the patients was LTFU. Of the 35 patients who died, 23 (65.7%) died in the first 6 months of treatment with a median time to death of 4 months (IQR: 2–9 months). Those patients who were declared cured, the median duration of treatment was 21 months (IQR: 21–24 months).

In MVBLR analysis, after adjusting for history of treatment with SLD and use of amikacin, the use of ethambutol emerged as the only variable which had statistically significant negative association with unsuccessful outcome (OR = 0.36, 95% CI 0.14–0.89, p-value = 0.02). This model fit was based on a non-significant Hosmer Lemeshow (Chi-square = 0.50, p-value = 0.77) and overall percentage of 80.8% from classification table (Table 4). Out of 15 patients who were on STR, only one (6.5%) developed unsuccessful outcome vs 38/198 (19.2%) who were on LTR. Furthermore, all six patients (100%) who were on *bedaquiline* containing regimen achieved successful outcomes. Cross-tabulation between death and patients’ sociodemographic, microbiological and clinical characteristics is given in Additional file 1: Table S2. The percentage of death in patients who received ethambutol (8.3%) was significantly (p-value < 0.02) lower than those who did not receive it (20.6%).

**Table 4:** Factors associated with unsuccessful outcomes

Variable	Unsuccessful outcomes No. (%)	Univariate analysis OR (95% CI)	p-value	Multivariate analysis OR (95% CI)	p-value
Gender					
Female	28 (17.5)	Referent			
Male	11 (20.8)	1.23 (0.57–2.69)	0.59		
Age (years)					
< 5	3 (21.4)	Referent			
5–9	5 (20.8)	1.04 (0.21–5.19)	0.96		
10–14	31 (17.8)	1.27 (0.33–4.81)	0.72		
Baseline body weight					
Normal	15 (15.6)	Referent			
Below normal	24 (20.5)	1.39 (0.68–2.84)	0.36		
Co-morbidity					
No	37 (18.7)	Referent			
Yes	2 (13.3)	0.67 (0.14–3.09)	0.60		
Previous TB treatment					
No	15 (19.0)	Referent			
Yes	23 (18.4)	0.96 (0.47–1.98)	0.91		
Unknown	1 (11.1)	0.53 (0.06–4.59)	0.56		
History of treatment with SLD					
No	36 (17.6)	Referent		Referent	
Yes	3 (37.5)	2.82 (0.64–12.32)	0.16	2.70 (0.55–13.29)	0.22
Type of drug-resistant TB					
Rifampicin resistant	17 (20.2)	Referent			
Multidrug resistant	22 (17.1)	0.81 (0.40–1.64)	0.55		
Site of DR-TB					
Ex-PTB	4 (15.4)	Referent			
Pulmonary TB	35 (18.7)	1.27 (0.41–3.91)	0.68		
Sputum smear grading					
Negative	6 (13.0)	Referent			
Scanty (1–9 AFB/100 HPF), + 1 (10–99 AFB/100 HPF)	18 (19.8)	1.64 (0.60–4.47)	0.33		
+ 2 (1–9 AFB/HPF), + 3 (> 9 AFB/100 HPF)	15 (19.7)	1.64 (0.59–4.58)	0.34		
Number of resistant drugs					
1	17 (20.5)	Referent			
2–4	14 (15.6)	0.71 (0.33–1.56)	0.40		
> 4	8 (20.0)	0.97 (0.38–2.49)	0.95		
Resistant to all five first FLD					
No	33 (17.9)	Referent			
Yes	6 (20.7)	1.19 (0.45–3.16)	0.72		
Resistance to pyrazinamide					
No	24 (16.6)	Referent			
Yes	15 (22.1)	1.43 (0.69–2.93)	0.33		
Resistance to ethambutol					
No	26 (14.4)	Referent			
Yes	13 (24.1)	1.62 (0.76–3.44)	0.20		
Resistance to any SLD					
No	30 (18.4)	Referent			
Yes	9 (18.0)	0.97 (0.43–2.22)	0.94		
Resistance to fluoroquinolone					
No	30 (17.9)	Referent			
Yes	9 (20.0)	1.15 (0.50–2.64)	0.74		
Resistance to ethionamide					
No	37 (17.8)	Referent			
Yes	2 (40.0)	3.08 (0.49–19.09)	0.22		
Treatment strategy					
Shorter treatment regimen	1 (6.7)	Referent			
Longer treatment regimen	38 (19.2)	3.32 (0.42–26.07)	0.25		
Use of isoniazid					
No	36 (18.9)	Referent			
Yes	3 (3.0)	0.64 (0.18–2.28)	0.49		
Use of ethambutol					
No	32 (22.7)	Referent		Referent	
Yes	7 (9.7)	0.37 (0.153–0.89)	0.02	0.36 (0.14–0.89)	0.02
Use of amikacin					
No	9 (32.1)	Referent		Referent	
Yes	30 (16.2)	0.41 (0.17–0.99)	0.04	0.51 (0.20–1.28)	0.15
Use of capreomycin					
No	37 (18.6)	Referent			
Yes	2 (16.7)	0.89 (0.19–4.22)	0.88		
Use of levofloxacin					
No	9 (16.1)	Referent			
Yes	30 (19.1)	1.23 (0.54–2.79)	0.61		
Use of moxifloxacin					
No	31 (19.5)	Referent			
Yes	8 (14.8)	0.72 (0.31–1.67)	0.44		
Use of para-amino salicylic acid					
No	23 (16.1)	Referent			
Yes	16 (22.9)	1.55 (0.76–3.16)	0.23		
Use of linezolid					
No	32 (17.3)	Referent			
Yes	7 (25.0)	1.59 (0.62–4.06)	0.32		
Use of bedaquiline					
No	39 (18.8)	Referent			
Yes	–	Non-computable			
Use of clofazimine					
No	36 (18.7)	Referent			
Yes	3 (15.0)	1.59 (0.62–4.06)	0.32		
Use of delamanid					
No	38 (18.2)	Referent			
Yes	1 (25.0)	1.50 (0.15–14.82)	0.72		

*AFB* acid fast bacilli, *DR-TB* drug-resistant tuberculosis, *FLD* first line anti-TB drugs, *HPF* high power field, *SLD* second line anti-TB drugs

**Fig. 1:**
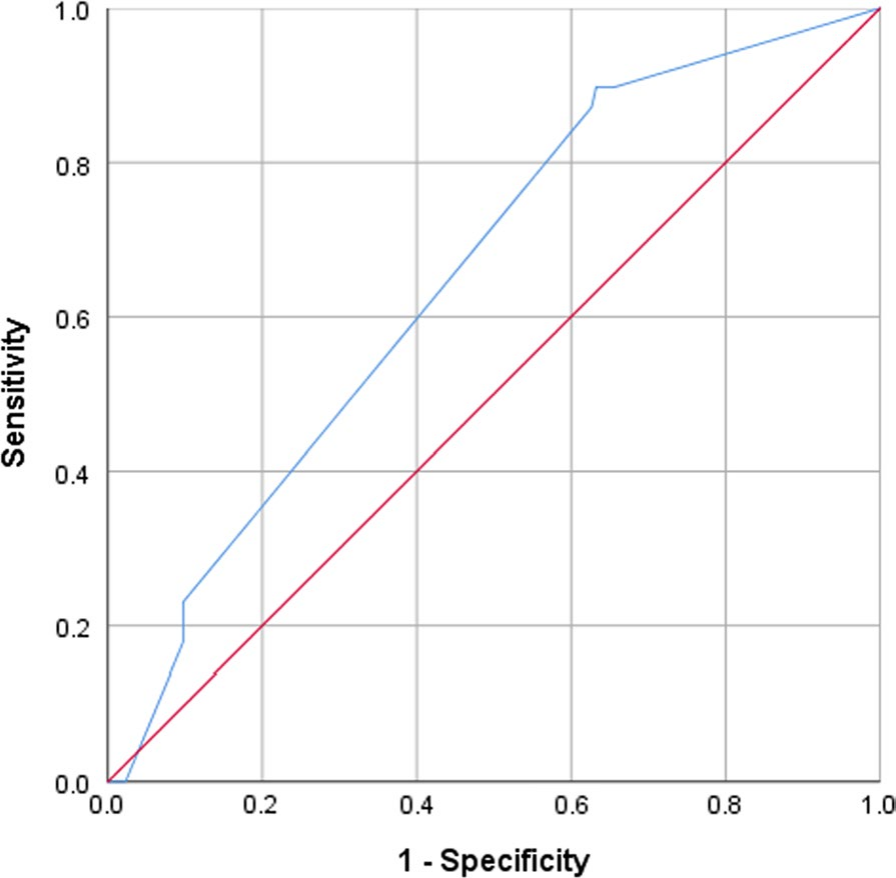
ROC curve of discriminatory power of final model predicting unsuccessful outcomes

However, the ROC curve analysis revealed poor discrimination power of the final model (AUC = 0.65, 95% CI 0.56–0.74, p-value = 0.003) (Fig. 1).

## Discussion

To the best of our knowledge, this is the first study which has evaluated the socio-demographic characteristics, drug resistance pattern, treatment outcomes and factors associated with unsuccessful outcomes of an individual cohort of childhood RR/MDR-TB in Pakistan, an MDR-TB 5th high burden country. In compliance with reports from Peru [9] and India [10], majority (74.2%) of the current study participants belonged to the age group of 11–14 years. The small proportions of children of age ≤ 5 years (8.9%) and 6–10 years (16.9%) in the current study and similar findings elsewhere [9, 10] highlights the known difficulties in the diagnoses childhood DR-TB which include the younger children incapability to expectorate sputum for culture and DST, and paucibacillary nature of the disease in these patients [10]. In our study, the disproportionately high number of female patients (74%) was inconsistent with the reported global epidemiology of TB in which male gender predominates [2]. However, it was in line with few reports from Pakistan [17, 19] and India [10] in which the proportion of female MDR-TB patients was disproportionately high. Upon cross-tabulation, we found that 140/160 female childhood patients of the current cohort were 10–14 years old. As common in poor communities, adult women and girls of this age take care of people and patients at home, this perhaps make them more susceptible to contract the infectious diseases. Furthermore, in Pakistan due to deeply rooted gender discrimination and TB related stigma, female TB patients suffer from greater delay in seeking healthcare and seek low quality care. This in turn may result in faulty diagnosis, guidelines divergent practices of healthcare providers, patients’ poor adherence with TB treatment regimen TB [25], and the development of DR-TB [19, 30]. In the current study, 84 patients (39.4%) suffered from RR-TB. However, only 20/84 (23.8%) of these patients were phenotypically confirmed. In the remaining 64 (76.4%) patients, the diagnosis of RR-TB was based on the results of Gene-Xpert, which could be one of the possible reasons for high proportion of RR-TB patients in the current cohort.

The high proportion of current patients with no history of previous TB treatment (37.1%) was consistent with the recent reports from Pakistan [22, 26] and elsewhere [10, 31, 32]. This suggests that primary transmission is becoming a major mode of spreading DR-TB in both adults and children and needs urgent measures of infection control to halt its spread [22, 31, 32]. In this study, only 50 (23.1%) patients were resistant to any SLD of whom 49 were MDR and one was RR-TB patient. Out of these 50 SLD resistant patients, 45 were resistant to FQ. In this study, 38% of MDR-TB patients were resistant to any SLD. This was lower than the range (41.3–55.5%) reported among MDR-TB patients (children and adults combined) in Pakistan [15, 19, 25, 33]. Prolonged delays between onset of TB symptoms and presentation to TB treatment centers, self-medication of chest symptomatics prior to TB diagnosis, treatment by inadequately aware local paramedics and private practitioners with insufficient diagnostic facilities, liberal use of pharmacy driven broad spectrum fluoroquinolones for respiratory tract infections, doctors non-compliance with TB treatment guidelines and patients non-adherence with TB treatment regimen have been reported as some of the major reasons of development of SLD resistance in DR-TB patients [25]. As a notable proportion of patients (37.1%) had no history of TB treatment, this could be one of the possible reasons of comparatively lower prevalence of SLD resistance in MDR-TB patients in this study.

The currently observed rate of sputum culture conversion (85.4%) among PTB patients was comparable with a study conducted in India (88%) [10]. However, median time to sputum culture conversion in our study (2 months, IQR: 1–3 months) was relatively shorter than what was observed in the Indian study (3 months, IQR: 3–4 months) [10]. Furthermore, in the current cohort, 89.8% of the patients who achieved SCC were culture negative by third month of treatment as compared to 73% in the Indian study [10].

The treatment success rate (81.7%) in the current cohort was above the target set by WHO (> 75%) and success rates observed among children and adolescent MDR-TB patients in India (62%) [10], children MDR-TB patients in Peru (77.3%) [9] and pooled treatment success rate of childhood MDR-TB (73%) in developing countries [11]. Furthermore, it was above the success rates (range: 40.5–76.9%) reported among MDR-TB patients (adults and children combined) treated with LTR in Pakistan [17–21]. However, it was lower than the success rate observed among childhood MDR-TB patients in South Africa (92%) [6] and the pooled treatment success rate observed in developed countries (87%) [11]. A total of 35 (16.4%) participants of this study died. This was consistent with the death rate (16%) among children and adolescent MDR-TB patients reported from India [10], but above the rates reported from Peru (4.3%) [9], by a meta-analysis of 1343 childhood MDR-TB patients (8%) [11] and a study from South Africa (2%) [6]. In our study, none of the participants was LTFU. The comparatively lower mortality rate in aforementioned studies could be due the masking of deaths by high comparatively high LTFU rates in these studies (range: 5–13.7%) [6, 9, 11]. In the current study, no significant difference in treatment success rate was observed between RR and MDR-TB patients. However, out of 15 patients who were on STR, only one (6.5%) developed unsuccessful outcome vs 38/198 (19.2%) who were on LTR. But due to its use in limited number of patients it did not achieve the level of significance in the model predicting treatment outcomes. In this study, all six patients (100%) who were on *bedaquiline* containing regimen achieved successful outcomes. The use of *bedaquiline* containing regimen has previously been reported to produce high treatment success rate and decrease in mortality among DR-TB patients [1], therefore, it has recently been included in group A anti-TB core drugs, recommended by WHO [16] and adopted by NTP as an integral component of DR-TB treatment regimens for eligible DR-TB patients of age 6 years and above. However, similar to STR, *bedaquiline* containing regimen was received by a fraction of the current study participants (2.8%). The *bedaquiline* containing regimens for eligible DR-TB patients in Pakistan was initially introduced at 6 PMDT sites in 2016 and then expanded to all PDMT sites. As now after the recommendations of WHO, all oral STR containing *bedaquiline* has been adopted by all PMDT sites in the country [23], therefore, it is suggested to evaluate its effectiveness in Pakistani settings. In multivariable analysis, the use of ethambutol emerged as the only predictor of treatment outcomes. Patients who were using ethambutol were significantly less likely to develop unsuccessful outcomes than their counterparts. In the treatment of DR-TB ethambutol is not used as a core drug but a companion drug to prevent the acquisition of additional drug resistance. In the published literature, the use of ethambutol has not been reported as a predictor of successful outcomes in DR-TB patients. Furthermore, our finding of ethambutol as a predictor of successful outcome should be interpreted with the poor discrimination power of the final model visualized by the ROC curve analysis (AUC = 0.651, 95% CI 0.562–0.740, p-value = 0.003) (Fig. 1). Patients’ age, use of second-line injectable anti-TB drugs, high dose isoniazid and malnutrition which have previously been reported as predictors of treatment outcomes among childhood MDR-TB patients [6, 10, 12] were not significantly associated with treatment outcomes in the current study.

Large number of microbiologically diagnosed RR/MDR-TB patients from multiple centers is the major strength of the current study. However due to retrospective nature of data collection, the lack of information about chest radiography to document the extent and severity of pulmonary disease which has previously been reported as a predictor of unsuccessful treatment outcomes in children MDR-TB patients [6, 9], lack of information about adverse events and their impact on treatment outcomes and the absence of post-treatment follow-up to ensure the absence of relapses among children with treatment success are the major limitations associated with this study.

## Conclusions

In conclusion, our findings suggest that study sites collectively achieved the WHO target of successful treatment outcomes (> 75%) among childhood RR/MDR-TB patients. The notable percentage of patients with no history of previous TB treatment and the disproportionately high number of female DR-TB patients in the current cohort stress for infection control measures and provision early and high quality care of drug susceptible TB in female patients. Furthermore, the finding of ethambutol as a predictor of successful treatment outcomes needs further investigation in large number of childhood RR/MDR-TB patients.

## Supplementary Information

**Additional file 1: Table S1.** Treatment regimen. **Table S2.** Cross-tabulation between death and patients’ sociodemographic and clinical characteristics.

## Data Availability

All data gathered or analyzed during this study are included in the article. The raw data on which conclusions of this manuscript is based is available upon request. Please contact Nafees Ahmad at nafeesuob@gmail.com.

## Abbreviations

Am: Amikacin
AUC: Area under curve
Bdq: Bedaquiline
Cfz: Clofazimine
CI: Confidence interval
Cm: Capreomycin
Cs: Cycloserine
Dlm: Delamanid
DR-TB: Drug-resistant tuberculosis
DST: Drug susceptibility testing
E: Ethambutol
Eto: Ethionamide
ENRS: Electronic nominal recording and reporting system
FQ: Fluoroquinolone
H: Isoniazid
IQR: Interquartile range
Km: Kanamycin
LTR: Longer treatment regimen
LTFU: Loss to follow up
Lzd: Linezolid
MDR-TB: Multi DR-TB
NTP: National TB Control Program
OR: Odds ratio
PAS: Para-amino salicylic acid
PMDT: Programmatic management of DR-TB
R: Rifampicin
ROC: Random operating characteristic curve
SCC: Sputum culture conversion
STR: Shorter treatment regimen
WHO: World Health Organization
Z: Pyrazinamide
