# Supplementary material for: High rate of successful treatment outcomes among childhood rifampicin/multidrug-resistant tuberculosis in Pakistan: a multicentre retrospective observational analysis
Source: BMC Infect Dis. 2021 Dec 4;21:1209. doi: 10.1186/s12879-021-06935-6 (PMC8642947; doi:10.1186/s12879-021-06935-6)
Supplement: Supplementary file 1 — Additional file 1: Table S1. Treatment regimen. Table S2. Cross-tabulation between death and patients’ sociodemographic and clinical characteristics. [file 12879_2021_6935_MOESM1_ESM.docx]

**Table S1: Treatment regimen**

| **Treatment regimen** | **No. (%)** |
| --- | --- |
| FQ+Eto+Cs+Z+Lzd/FQ+Eto+Cs+Z+Lzd  SLI+FQ+Eto+Cs+Z+Lzd/FQ+Eto+Cs+Z+Lzd  SLI+FQ+Eto+Cs+Z+Lzd+E+Bdq(6)/FQ+Eto+Cs+Z+Lzd+E  SLI+FQ+Eto+Cs,Z+E+H/FQ+Eto+Cs+Z+E+H  FQ+Eto+Cs+Z+Lzd+H+E/FQ+Eto+Cs+Z+Lzd+H+E  SLI+FQ+Eto+Cs+Cfz+Bdq(6)/FQ+Eto+Cs+Cfz  SLI+FQ+Eto+Cs+PAS+Z+Aug+Clr/ FQ+Eto+Cs+PAS+Z+Aug+Clr  SLI+FQ+ETO+CS+PAS+Z+Cfz+Aug/ FQ+ETO+CS+PAS+Z+Cfz+Aug  SLI+FQ+Eto+Cs+PAS+Z+Cfz+Lzd+Aug+Clr+Bdq(6)+Dlm/  FQ+Eto+Cs+PAS+Z+Cfz+Lzd+Aug+Clr+Dlm  SLI+FQ+Eto+Cs+PAS+Z+Cfz+Lzd+E+Aug/  FQ+Eto+Cs+PAS+Z+Cfz+Lzd+E+Aug  SLI+FQ+Eto+Cs+PAS+Z+E+H/FQ+Eto+Cs+PAS+Z+E+H  SLI+FQ+Eto+Cs+PAS+Z+Lzd+E/FQ+Eto+Cs+PAS+Z+Lzd+E  SLI+FQ+Eto+CS+Z+Cfz/FQ+Eto+CS+Z+Cfz  SLI+FQ+Eto+Cs+Z,Lzd+E+Aug/FQ+Eto+Cs+Z,Lzd+E+Aug  SLI+FQ+Eto+Cs+Z+Lzd+E/FQ+Eto+Cs+Z+Lzd+E  SLI+FQ+Eto+Cs+Z+Lzd+E+Dlm(6)/FQ+Eto+Cs+Z+Lzd+E  SLI+FQ+Eto+Z+Cfz+Dlm(6)/FQ+Eto+Z+Cfz  SLI+FQ+Eto+Z+Cfz+E/FQ+Eto+Z+Cfz+E  SLI+FQ+Eto+Z+Lzd/FQ+Eto+Z+Lzd  SLI+FQ+Eto+Z+R+H/FQ+Eto+Z+R+H | 7 (3.3)  6 (2.8)  4 (1.9)  2 (0.9)  1 (0.5)  1 (0.5)  1 (0.5)  1 (0.5)  1 (0.5)  1 (0.5)  1 (0.5)  1 (0.5)  1 (0.5)  1 (0.5)  1 (0.5)  1 (0.5)  1 (0.5)  1 (0.5)  1 (0.5)  1 (0.5) |

Aug: Co-amoxiclav. Cfz: clofazimine, Clr: clarithromycin, Cs: cycloserine, Dlm: delamanid; E: ethambutol, Eto: ethionamide, FQ: fluoroquinolones, H: isoniazid, Km: kanamycin, Lzd: Linezolid, PAS: para-amino salicylic acid, R: Rifampicin, SLI: second-line injectable (amikacin/kanamycin/capreomycin), Z: pyrazinamide

**Table S2: Cross-tabulation between death and patients’ sociodemographic and clinical characteristics**

| **Variables** | **Died** No. (%)  No Yes | | **p-value** |
| --- | --- | --- | --- |
| **Gender**  Female  Male | 136 (85.0)  42 (79.2) | 24 (15.0)  11 (20.8) | 0.32 |
| **Age** (years)  <5  5-9  10-14 | 11 (78.6)  19 (79.2)  148 (84.6) | 3 (21.4)  5 (20.8)  27 (15.4) | 0.579* |
| **Co-morbidity**  No  Yes | 165 (83.3)  13 (86.7) | 33 (16.7)  2 (13.3) | 1.00* |
| **Previous TB treatment**  No  Yes  Unknown | 65 (82.3)  105 (84.0)  8 (88.9) | 14 (17.7)  20 (16.0)  1 (11.1) | 0.86 |
| **History of treatment with SLD**  No  Yes | 172 (83.9)  6 (75.0) | 33 (16.1)  2 (25.0) | 0.62* |
| **Type of drug-resistant TB**  Rifampicin resistant  Multidrug resistant | 68 (81.0)  110 (85.3) | 16 (19.0)  19 (14.7) | 0.41 |
| **Site of DR-TB**  Ex-PTB  Pulmonary TB | 22 (84.6)  156 (83.4) | 4 (15.4)  31 (16.6) | 1.00* |
| **Sputum smear grading**  Negative  Scanty(1–9 AFB/100 HPF),+1 (10–99 AFB/100 HPF)  +2 (1–9 AFB/HPF), +3 (>9 AFB/100 HPF) | 42 (91.3)  73 (80.2)  63 (82.9) | 4 (8.7)  18 (19.8)  13 (17.1) | 0.25 |
| **Number of resistant drugs**  1  2-4  >4 | 66 (79.5)  78 (86.7)  34 (85.0) | 17 (20.5)  12 (13.3)  6 (15.0) | 0.43 |
| **Resistant to all five FLD**  No  Yes | 154 (83.7)  24 (82.8) | 30 (16.3)  5 (17.2) | 1.00* |
| **Resistance to pyrazinamide**  No  Yes | 122 (84.1)  56 (82.4) | 23 (15.9)  12 (17.6) | 0.74 |
| **Resistance to ethambutol**  No  Yes | 134 (84.3)  44 (81.5) | 25 (15.7)  10 (18.5) | 0.63 |
| **Resistance to streptomycin**  No  Yes | 140 (82.4)  38 (88.4) | 30 (17.6)  5 (11.6) | 0.34 |
| **Resistance to any SLD**  No  Yes | 135 (82.8)  43 (86.0) | 28 (17.2)  7 (14.0) | 0.59 |
| **Resistance to fluoroquinolone**  No  Yes | 140 (83.3)  38 (84.4) | 28 (16.7)  7 (15.6) | 0.58 |
| **Resistance to ethionamide**  No  Yes | 175 (84.1)  3 (60.0) | 33 (15.9)  2 (40.0) | 0.19 |
| **Treatment strategy**  Shorter treatment regimen  Longer treatment regimen | 14 (93.3)  164 (82.8) | 1 (6.7)  34 (17.2) | 0.47* |
| **Use of isoniazid**  No  Yes | 158 (83.2)  20 (87.0) | 32 (16.8)  3 (13.0) | 0.77* |
| **Use of ethambutol**  No  Yes | 112 (79.4)  66 (91.7) | 29 (20.6)  6 (8.3) | 0.02 |
| **Use of amikacin**  No  Yes | 21 (75.0)  157 (84.9) | 7 (25.0)  28 (15.1) | 0.18 |
| **Use of capreomycin**  No  Yes | 167 (83.1)  11 (91.7) | 34 (16.9)  1 (8.3) | 0.69 |
| **Use of levofloxacin**  No  Yes | 48 (85.7)  130 (82.8) | 8 (14.3)  27 (17.2) | 0.61 |
| **Use of moxifloxacin**  No  Yes | 131 (82.4)  47 (87.0) | 28 (17.6)  7 (13.0) | 0.42 |
| **Use of para-amino salicylic acid**  No  Yes | 122 (85.3)  56 (80.0) | 21 (14.7)  14 (20.0) | 0.32 |
| **Use of linezolid**  No  Yes | 157 (84.9)  21 (75.0) | 28 (15.1)  7 (25.0) | 0.18* |
| **Use of bedaquiline**  No  Yes | 172 (83.1)  6 (100) | 35 (16.9)  - | 0.59* |
| **Use of clofazimine**  No  Yes | 161 (83.4)  17 (85.0) | 32 (16.6)  3 (15.0) | 1.00* |
| **Use of delamanid**  No  Yes | 175 (83.7)  3 (75.0) | 34 (16.3)  1 (25.0) | 0.51* |

AFB: acid fast bacilli, DR-TB: drug-resistant tuberculosis, FLD: first-line anti-TB drugs, HPF: high power field, SLD: second-line anti-TB drugs

*Fisher-exact test
